# The first complete chloroplast genome of *Haymondia wallichii* (Fabaceae) and its phylogenetic analysis

**DOI:** 10.1080/23802359.2021.1923415

**Published:** 2021-06-07

**Authors:** Xianjun Huang, Conglong Xia, Yuan Zhao, Li Xu

**Affiliations:** a College of Pharmaceutical Science, Dali University, Dali, China; b College of Nursing, Dali University, Dali, China; c College of Basic Medicine, Dali University, Dali, China; d Key Laboratory of Yunnan Provincial Higher Education Institutions for Development of Yunnan Daodi Medicinal Materials Resources, Dali, China

**Keywords:** *Haymondia wallichii* DC., complete chloroplast genome, phylogenetic analysis

## Abstract

*Haymondia wallichii* DC. was a scandent shrub. In this study, we sequenced the complete chloroplast (cp) genome of *H. wallichii* to investigate its phylogenetic relationship in Fabaceae. The total length of the cp genome was 153,668 bp, consisted of a large single copy (LSC) region of 84,310 bp, a small single copy (SSC) region of 17,918 bp, and a pair of inverted repeat regions (IRs) of 25,720 bp. The genome contained 132 genes, namely 37 tRNA genes, 87 protein-coding genes, and 8 rRNA genes. The overall GC content was 35.4%. The phylogenetic analysis indicated *H. wallichii* was closely related to *Pueraria montana* var. *thomsonii a*nd *Pueraria montana* var. *lobata.*

*Haymondia wallichii* DC. is a species belonging to the family Fabaceae, which is mainly distributed in Yunnan and Tibet in China. *H. wallichii* is a common traditional Chinese medicine for anti-alcoholic and health care in Dali, Yunnan. In the previous study, *H. wallichii* was belonged to *Pueraria* (Egan and Pan 2015), while the classification of *H. wallichii* had always been controversial. Although Egan and Pan divided it into a new genus of *Haymondia* based on morphological characters and phylogenetic studies (Egan and Pan 2015; Egan et al. 2016), it was still incomplete in molecular biology research. The accurate identification of *H. wallichii* can ensure the quality of *H. wallichii* (Miao et al. 2019) and different molecular techniques may lead to different phylogenetic results (Zhang et al. 2020). Therefore, the study reported the complete chloroplast genome of the *H. wallichii*, and revealed its phylogenetic relationship with other Fabaceae plants to lay the foundation for future research of *Haymondia*.

In this study, The fresh leaves of *H. wallichii* were collected from Weishan mountain (N 25°53′71.57″, E 100°25′49.78″) of Dali, Yunnan province, China, and used as molecular materials. Meanwhile, voucher specimens with mature seeds were collected as the sample and deposited in the Herbarium of Medicinal Plants and Crude Drugs of the College of Pharmaceutical Science, Dali University (NO. XCL018). Total genomic DNA was extracted using the improved CTAB method (Doyle 1987) and sequenced by the Illumina Hiseq 2500 platform (Novogene, Tianjin, China). The raw data was filtered using Trimmomatic version 0.32 with default settings (Bolger et al. 2014). Then paired-end reads of clean data were assembled by GetOrganelle.py into circular contigs (Jin et al. 2018). The cp genome of *H. wallichii* was annotated in Geneious 9.1.4 (Kearse et al. 2012). A physical map of the cp genome was obtained through the web-based tool OGDraw version 1.2 (http://ogdraw.mpimp-golm.mpg.de/). Distributions of the simple sequence repeats (SSRs) were explored by the microsatellite search tool MISA (Thiel et al. 2003). The annotated cp genome sequences had been submitted to GenBank (accession number：MT797172.1).

The cp genome of *H. wallichii* was 153,668 bp in length, with 35.4% overall GC content, and exhibited a typical quadripartite structure, consisting of a large single copy (LSC) region of 84,310 bp, a small single-copy (SSC) region of 17,918 bp, and a pair of IRs (two inverted repeat regions) of 25,720 bp. It contained 132 genes, including 37 tRNA genes, 87 protein-coding genes, and 8 rRNA genes. A total of 88 simple sequence repeats (SSRs) with five types were detected in the cp genome, including 49 mononucleotide repeats, 28 dinucleotide repeats and 11 other types of SSR loci.

To reveal the phylogenetic relationship of the *H. wallichii.* A total of 18 cp genomes of Fabaceae were downloaded from NCBI database. The sequences were aligned by MAFFT v7.307 (Katoh and Standley 2013). Then the neighbour-joining (NJ) tree was established based on MEGA X (Kumar et al. 2018) and used *Aristolochia* of Aristolochiaceae as the outgroup (Figure 1). Numbers in the nodes were the bootstrap values from 1000 replicates. The result showed that the cp genome of *H. wallichii* was closed to *Pueraria montana* var. *thomsonii* and *Pueraria montana* var. *Lobata*. This study of *H. wallichii* may provide a valuable guide to the *Haymondia* future researches of systematics and phylogenetic.

**Figure 1.**
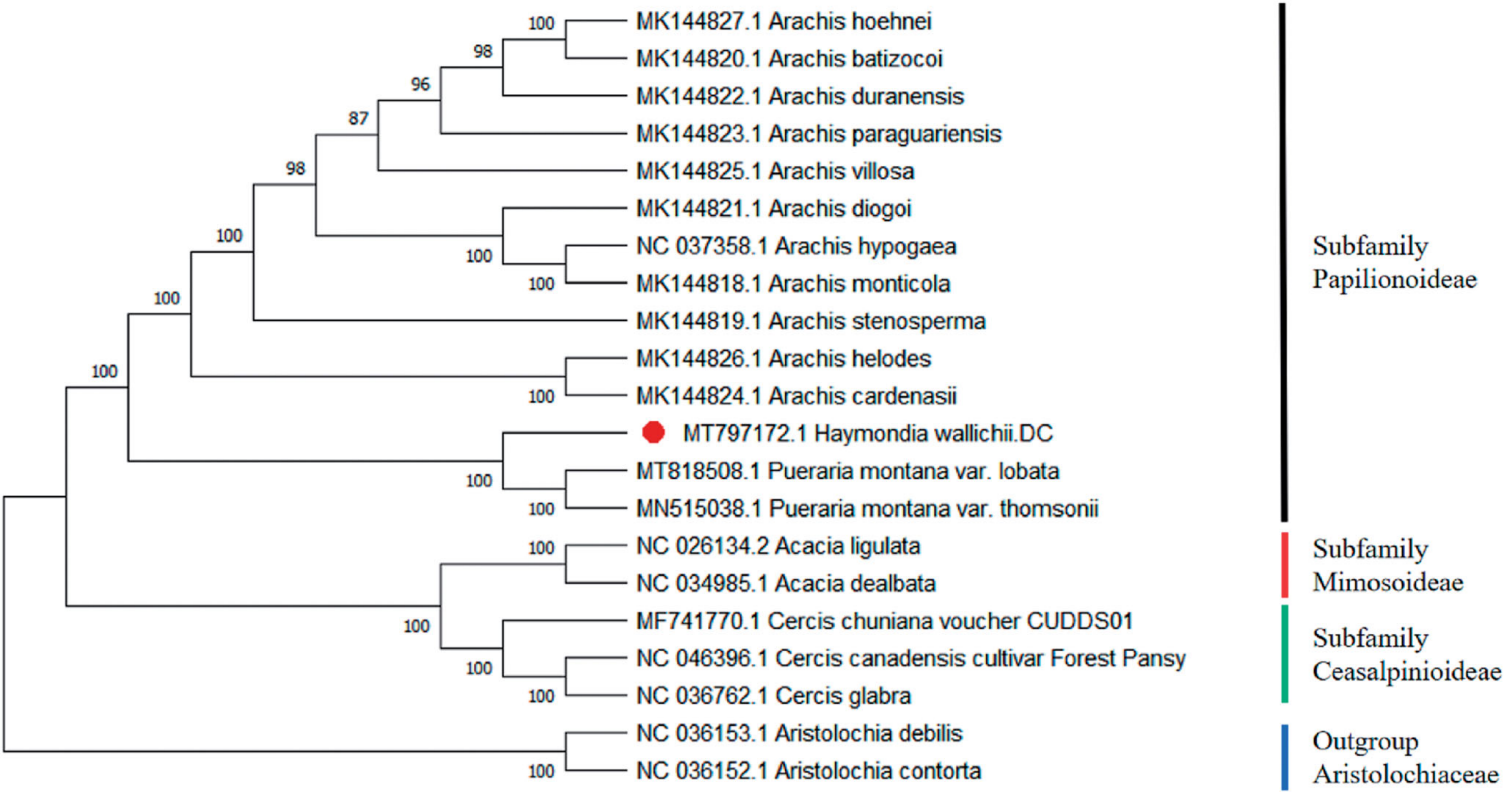
Phylogenetic position of *H. wallichii* inferred by the neighbour-joining (NJ) analysis based on 21 sequences, bootstrap values near the branch.

## Data Availability

The data that supports the findings of this study is openly available in NCBI GenBank database at (https://www.ncbi.nlm.nih.gov/nuccore/MT797172.1) with the accession number is MT797172.1, which permits unrestricted use, distribution, and reproduction in any medium, provided the original work is properly cited.
